# On the Transmission Dynamics of SARS-CoV-2 in a Temperate Climate

**DOI:** 10.3390/ijerph18041660

**Published:** 2021-02-09

**Authors:** Ioannis Kioutsioukis, Nikolaos I. Stilianakis

**Affiliations:** 1 Department of Physics, University of Patras, 26504 Rio, Greece; kioutio@upatras.gr; 2 Joint Research Centre (JRC), European Commission, 2027 Ispra, Italy; 3 Department of Biometry and Epidemiology, University of Erlangen-Nuremberg, 91054 Erlangen, Germany

**Keywords:** asymptomatic infections, temperature, humidity, SARS-CoV-2, COVID-19, predictability, transmission, epidemiological model

## Abstract

An epidemiological model, which describes the transmission dynamics of SARS-CoV-2 under specific consideration of the incubation period including the population with subclinical infections and being infective is presented. The COVID-19 epidemic in Greece was explored through a Monte Carlo uncertainty analysis framework, and the optimal values for the parameters that determined the transmission dynamics could be obtained before, during, and after the interventions to control the epidemic. The dynamic change of the fraction of asymptomatic individuals was shown. The analysis of the modelling results at the intra-annual climatic scale allowed for in depth investigation of the transmission dynamics of SARS-CoV-2 and the significance and relative importance of the model parameters. Moreover, the analysis at this scale incorporated the exploration of the forecast horizon and its variability. Three discrete peaks were found in the transmission rates throughout the investigated period (15 February–15 December 2020). Two of them corresponded to the timing of the spring and autumn epidemic waves while the third one occurred in mid-summer, implying that relaxation of social distancing and increased mobility may have a strong effect on rekindling the epidemic dynamics offsetting positive effects from factors such as decreased household crowding and increased environmental ultraviolet radiation. In addition, the epidemiological state was found to constitute a significant indicator of the forecast reliability horizon, spanning from as low as few days to more than four weeks. Embedding the model in an ensemble framework may extend the predictability horizon. Therefore, it may contribute to the accuracy of health risk assessment and inform public health decision making of more efficient control measures.

## 1. Introduction

Several questions surround the transmission dynamics of Severe Acute Respiratory Syndrome-related Corona Virus 2 (SARS-CoV-2), which is the causative agent of Corona Virus Disease 2019 (COVID-19). A characteristic of epidemiological importance for COVID-19 is the existence of a state of subclinical infection during which an infected individual does not show clinical symptoms but can spread the infection to other individuals [1]. The contribution of these, so called, asymptomatic individuals (subclinical infections) to the transmission of SARS-CoV-2 has not been well understood. Recent virological and epidemiological modelling studies indicate that a significant fraction of infected people are asymptomatic with the potential of person-to-person transmission. Epidemiological evidence emerged predominantly from household studies [2,3,4,5]. Virological studies showed that infected people who recovered without developing symptoms had moderate or high viral loads implying rather high infectiousness during subclinical infections [6,7,8]. Epidemiological modelling studies estimated the serial interval (time between symptom onset in a primary patient and symptom onset in the secondary patient) to be close to the incubation period that may indicate transmission before the development of symptoms [9,10]. Some approaches provided an estimate of the ability of asymptomatic infections to transmit the pathogen [11] and the fraction of asymptomatic infections to be in the range of about one fifth and more than half among all infected persons observed [12,13,14]. These studies imply that the speed and extent of SARS-CoV-2 transmission cannot be accounted for solely by transmission from infected persons with clinical symptoms. The epidemiological significance of this population group needs to be addressed to understand to what extent subclinical infections drive the epidemic and their relative importance compared to individuals who develop clinical symptoms (illness). If this fraction of infected persons is substantial, the case fatality risk for COVID-19 may be lower than currently estimated [15]. Moreover, it also implies that control of the epidemic may be more difficult since pathogen transmission from asymptomatic infected persons may be a non-negligible source of transmission.

Any intervention strategy aimed at controlling the outbreak depends strongly on the ability to identify infected persons which is difficult to identify with the absence of symptoms [16]. This is particularly important for newly emerging pathogens (e.g., SARS-CoV-2, pandemic influenza viruses) due to the lack or limited availability of effective drugs or vaccines. Public health responses rely on early estimates of crucial epidemiological characteristics, such as the case fatality risk and the transmissibility expressed as the basic reproduction number, which is usually derived from epidemiological models or statistically estimated from epidemiological data [17,18,19]. However, the existence of subclinical infections leads to enormous challenges of estimating these quantities. The consequences are uncertainty in epidemiological data and models, impairment of the public health decision makers and difficulties in assessing the effectiveness of proposed interventions. Asymptomatic infected individuals can have a critical impact on the effectiveness of non-pharmaceutical interventions such as social distancing [20].

**Figure 1:**
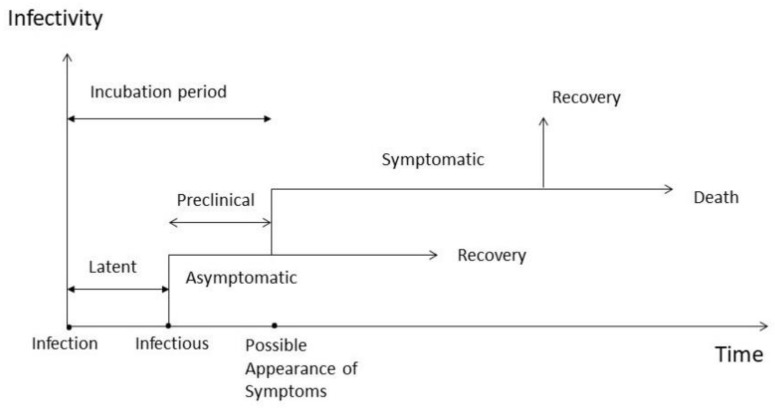
Timeline for infection and disease.

Two fundamental approaches have been used to embed subclinical infections in epidemiological models. The first divides the infectious population into two states immediately following the onset of infectiousness, namely asymptomatic and symptomatic persons [21,22,23]. This approach ignores or considers as negligible the latent period and the preclinical infectious period, together representing the incubation period. A biologically more plausible pathway for diseases such as influenza and in particular COVID-19 that has a long incubation period is a model that allows for the latent period and preclinical state, which is an asymptomatic infectious state preceding the onset of symptoms (Figure 1). This approach has been used in the epidemiological modelling of influenza [24,25,26,27,28]. The latent period is defined as the initial period after infection where an infected person is infected but non-infectious yet. A mathematical elaboration of the similarities and differences of the two models is presented in [28].

Virus inactivation in the environment is of high relevance for the relative importance of modes of transmission of respiratory viruses and epidemic control. The roles of temperature and humidity have been addressed in the past predominantly for influenza [29] and also recently for SARS-CoV-2 [30]. This also applies to the role of environmental ultraviolet radiation and low indoor humidity [29,31]. Many respiratory viruses such as influenza occur during the winter months in the Northern and Southern Hemisphere suggesting that the winter climatic conditions may play a role in the spread of respiratory virus infections [32,33]. Seasonal determinants and host factors affected by seasonal factors seem to be involved [34,35,36,37]. Changes in environmental factors (such as temperature, absolute humidity, sunlight), viral factors and host immunity and behavioral factors, household crowding indoors during cold weather leading to increased contact rates to name a few, may be relevant, all acting and having an effect individually and most probably in combination [38,39,40]. For epidemics occurring in the winter season, temperature and humidity have been identified as potential drivers of seasonality of respiratory infectious agents. It has been hypothesized that this might be the case for SARS-CoV-2 too [41,42,43,44,45].

In this paper, we extended a model previously developed by one of the authors that explicitly incorporated subclinical infections to account for the contribution of this population group to the transmission dynamics of respiratory pathogens such as influenza and SARS-CoV-2 [24,28]. In the model, there are three classes of infected individuals; those who are in the initial latent period are infected but not infectious; those who are infectious and develop symptoms after a preclinical asymptomatic period, and those who remain asymptomatic but infectious and recover without passing through the symptomatic state. We explored the effects in the dynamics due to this distinction between asymptomatic individuals and those with clinical symptoms. We quantified the fractions of the infectious subpopulations and described their dynamic change with and without control measures that were exemplified in the case of social distancing. The effects of the relative difference in the transmission between these groups of infective individuals, and the recovery period in the overall disease dynamics were shown. A clustering of the occurrence and the number of COVID-19 human cases with respect to temperature and humidity was initially presented to assess whether substantial impact of climate effects on virus transmission exists. Then, applying a Monte Carlo uncertainty framework for error optimization in the model we estimated the values of the epidemiological parameters and their relationship in the case of the COVID-19 epidemic in Greece. Moreover, following the diagnostic evaluation, the model was also applied in predictive mode to investigate the intra-annual variability of the transmissivity and the predictability horizon.

## 2. Materials and Methods

### 2.1. The Model

Several studies have used epidemiological models to describe the transmission dynamics of SARS-CoV-2 within a susceptible population by also taking into account interventions [10,20,46,47,48]. Some of these studies also consider asymptomatic individuals being infectious [49,50,51]. Our approach differs by the way we modelled the states of the infection process, the specific focus being on the relationship between the transmission and recovery rates, and how they affect the dynamics using a Monte Carlo uncertainty analysis framework for the estimation of the parameters that can be tailored for any outbreak individually. For instance, we did not adapt the model parameters to account for the interventions arbitrarily, but we based the adaptation on the country specific best fit analysis framework. Finally, we also looked at the potential effects of climatic factors such as temperature and humidity.

**Figure 2:**
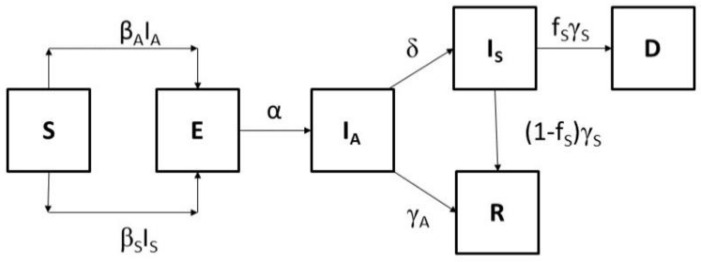
Transition diagram of model populations. Susceptible (*S*) persons become infected after exposure to respiratory droplets exhaled from asymptomatic infected persons (*I_A_*) or infected persons with clinical symptoms (*I_S_*) with rates *β_A_* and *β_S_*. Exposed and infected persons (*E*) go through a period of latency during which they are not infectious. Exposed persons become asymptomatic infected and infectious (*I_A_*) with a rate *α*. Asymptomatic infected persons develop clinical symptoms and become infected with symptoms (illness) with a rate *δ* or they recover (*R*) with a rate *γ_A_*. Persons with clinical symptoms can recover (*R*) with a rate (1 − *f_S_*) *γ_S_* or die (*D*) with a rate *f_S_γ_S_*.

The model consists of six distinct classes such that the total population is *N(t) = S(t) + E(t) + I_A_(t) + I_S_(t) + R(t) + D(t)*. *S(t)* denotes the number of susceptible individuals at time *t*, *E(t)* is the number of exposed (infected but not infectious) individuals, *I_A_(t)* is the number of asymptomatic infective individuals, *I_S_(t)* is the number of infected individuals with symptoms, *R(t)* is the number of individuals who recovered and were removed, and *D(t)* the number of fatalities during the outbreak. We assumed that the total population remains constant during the outbreak. The assumption of a constant population is reasonable when the infection spreads fast through the population, as this is the case for the COVID-19 pandemic. Therefore, we did not include demographic effects such as births and natural deaths in the model. The modelling approach also implies that the populations are homogeneously mixed. We also ignored high-risk groups. We explicitly included the characteristic incubation period between infection and the appearance of clinical symptoms. This period consists of the latency and the preclinical state (Figure 1). The pathogen is transmitted to a susceptible person through person-to–person contact (via generation of respiratory droplets containing infectious pathogens) with either an asymptomatic infected person or an infected person who developed clinical symptoms (illness). Susceptible individuals (*S*), once infected, enter the state of exposed individuals (*E*) harbouring a latent infection but not being infectious. All exposed individuals enter the state of being asymptomatic and having subclinical infections. Asymptomatic individuals (*I_A_*) are infectious and there are two options. An individual may remain asymptomatic for the duration of the infection and recover without ever developing clinical symptoms entering the recovery state (*R*). Alternatively, an individual may remain asymptomatic (preclinical) for a period of time before ultimately entering the clinical state and becoming an infected individual with clinical symptoms (*I_S_*). For those becoming ill with clinical symptoms a fraction recovers and some may die. We assumed that recovered individuals obtain immunity, and they are removed from the infection process. Figure 2 shows the transitions between the different states of the infection pathway. The equations of the model are written as follows:

(1) $$\frac{dS\left(t\right)}{dt}=-\beta_{A}\frac{I_{A}\left(t\right)S\left(t\right)}{N}-\beta_{S}\frac{I_{S}\left(t\right)S\left(t\right)}{N}$$

(2) $$\frac{dE\left(t\right)}{dt}=\beta_{A}\frac{I_{A}\left(t\right)S\left(t\right)}{N}+\beta_{S}\frac{I_{S}\left(t\right)S\left(t\right)}{N}-\alpha E\left(t\right)$$

(3) $$\frac{dI_{A}\left(t\right)}{dt}=\alpha E\left(t\right)-\left(\delta +\gamma_{A}\right)I_{A}\left(t\right)$$

(4) $$\frac{dI_{S}\left(t\right)}{dt}=\delta I_{A}\left(t\right)-\gamma_{S}I_{S}\left(t\right)$$

(5) $$\frac{dR\left(t\right)}{dt}=\gamma_{A}I_{A}\left(t\right)+(1-f_{S})\gamma_{S}I_{S}\left(t\right)$$

(6) $$\frac{dD\left(t\right)}{dt}=f_{S}\gamma_{S}I_{S}\left(t\right)$$

The initial conditions of the model are: *S*(0) = *S*_0_; *E*(0) = 0; *I_A_*(0) = *I_A_*_0_; *I_S_*(0) = *I_S_*_0_; *R*(0) = 0; *D*(0) = 0. One may start the epidemic with a few infected persons with clinical symptoms and/or asymptomatic individuals. The equation for the fatalities does not contribute to the dynamics of the system. It simply counts the number of fatalities.

Transmission of infection occurs with a rate and results from contacts of susceptible with infected persons. The transmission rate from an asymptomatic person to a susceptible is presumed to be lower (*β_A_*) than that to an infected person who develops symptoms (*β_S_*). The rationale behind this assumption is that as observed not every infection leads to illness. In addition, there is probably a difference, though difficult to quantify, in transmissibility between transmission from asymptomatic infected to susceptible persons and transmission from symptomatic infected to susceptible persons. An infected person with clinical illness sheds more virus that one with subclinical infection [52]. Moreover, a clinically ill person has symptoms (e.g., coughing, rhinorrhea) that contribute to the generation of infectious pathogen laden droplets of all sizes. On the other side, infected persons with clinical symptoms may show reduced virus transmissibility if they are confined to bed due to severity of illness. Therefore, the magnitude of difference between the two transmission rates depends on factors such as age, severity of illness, social behavior, living conditions in households (common rooms, share bedrooms) and others.

Transition from the exposed to the asymptomatic infected state occurs with a rate *α* indicating that the period between infection and development of infectiousness is 1/α. An asymptomatic infected individual moves from the asymptomatic infected state (*I_A_*) to the symptomatic infected state (*I_S_*) with a rate *δ* denoting the period from onset of infectiousness to onset of clinical symptoms and may remain asymptomatic (preclinical infection) for a period of *1/δ* before developing symptoms. Alternatively, an asymptomatic individual may never develop symptoms and recover after a period *1/γ_A_*. Therefore, the average time of an asymptomatic individual in state *I_A_* is *1/(δ + γ_A_)*. The incubation period is denoted as the period from infection to onset of symptoms (*1/α) + (1/δ).* Asymptomatic infected persons are expected to recover faster than those who have developed clinical symptoms. Symptomatic infected individuals are likely to have greater infectivity due to higher virus loads higher viral shedding due the severity of their clinical conditions. Thus, following the onset of symptoms, the recovery period for a person with symptoms is *1/γ_S_*. Finally, the fraction of persons who developed severe illness and died is denoted with *f_s_*.

### 2.2. The Basic Reproduction Number

The basic reproduction number (*R*_0_) represents the average number of secondary infections where an infected individual can cause in an entirely susceptible population. *R*_0_ can be calculated using the disease-free equilibrium of the above system of ordinary differential equations. It can be derived using the next generation matrix using the methods described in [53]. In this case the *R*_0_ is the sum of the basic reproduction number for the infections caused by an asymptomatic individual and that of an individual with symptoms.

(7) $$R_{0}=R_{A}+R_{S}=\beta_{A}\frac{S_{0}}{N\left(\delta +\gamma_{A}\right)}+\beta_{S}\frac{S_{0}\delta }{N\gamma_{S}\left(\delta +\gamma_{A}\right)}$$

For $S_{0}\approx N\left(0\right)$

(8) $$R_{0}=R_{A}+R_{S}=\frac{\beta_{A}}{\left(\delta +\gamma_{A}\right)}+\frac{\beta_{S}\delta }{\gamma_{S}\left(\delta +\gamma_{A}\right)}$$

Note that the basic reproduction number does not depend on the transition rate *α* from the exposed to the asymptomatic state. The *R*_0_ is a threshold. If $R_{0}>1$ then the number of infective individuals first increases before decreasing to zero representing a full-blown epidemic curve. If $R_{0}\;<1$ then the number of infected individuals decreases monotonically to zero.

### 2.3. The Monte Carlo Uncertainty Analysis and the Error Optimization

The parameter values we used in the model were based on estimates derived from epidemiological studies in different countries in the world affected by COVID-19 [54]. For each of the parameters we chose a range of possible values as these have been reported. The range represented prior distributions of the parameter values. We employed a Monte Carlo (MC) uncertainty framework for error optimization to derive the posterior distributions of the parameter values including their optimal values. This was implemented through the minimization of the quantity *NMSE(I_S_) + NMSE(D)*, where NMSE is the mean squared error (MSE) normalized with the mean of the corresponding observations (i.e., infected or dead). All parameters were optimized separately before, during and after the introduction of intervention, in this case social distancing in form of a population-based lockdown. Table 1 summarizes the range of prior distribution of the parameter values. We applied the model using the time series of cases as they were unfolded in Greece and as they were reported in the official web site of the national public health authority [55]. We run the simulations of the model with an initial population of susceptibles (*S*_0_ = 1 million people) and a small number of infected persons with clinical symptoms (*I_S_*_0_ = 1). Two cycles of Monte Carlo simulations were adopted. At the screening phase, we assumed the parameters followed a uniform distribution. The model simulations were compared with observations to identify the uncertainty range that minimized the error. At the implementation phase, the parameters were sampled from a normal distribution centred at their mode identified during the screening phase.

### 2.4. The Forecast Horizon

In public health decision making it is important to know the length of time into the future for which the model projections are below a certain error. We define the forecast horizon as the length of time into the future for which the divergence between the predicted (modelled) and the observed time series (infected humans or deaths or both) yields a NRMSE of 10%.

### 2.5. Clustering of COVID-19 Data

There have been indications that temperature and humidity may be associated with the transmission dynamics of SARS-CoV-2 [41,44,56]. For the exploration of the potential association of the SARS-CoV-2 transmission with climatic factors, we obtained all reported worldwide COVID-19 cases from the European Centre for Disease Prevention and Control (ECDC) (01/01/2020–07/05/2020) and the surface weather data for the same period at each country from the global weather database hosted at the University of Wisconsin [57]. For each country we matched the COVID-19 events (occurrence of human cases) and magnitudes (number of human cases) with the meteorological data of its capital, except for China and Italy where Wuhan and Milan were used instead. We divided the global COVID-19 cases into 25 groups according to their respective daily temperature (*T*) and absolute humidity (*ρ_v_*) and calculated the percentage of events within each group. We standardized the number of events within each cell with the number of weather records falling inside it (Probability of Infection). Further, we converted the magnitudes into number of human cases per million and estimated the median, to quantify the severity within each group.

## 3. Results

We performed initially 40,000 simulations using the time series of confirmed cases in Greece. We used uniform distributions for the uncertain factors in the first 20,000 simulations to narrow their range. Then, we applied a normal distribution in the second block of 20,000 simulations (MC1: 15/02/2020–31/05/2020). We started the simulations 10 days before the identification of the first case with clinical symptoms taking into account the incubation period and some delay in registration and reporting. The lockdown was introduced at day 37 (23/03/2020) and became ineffective at day 78 (04/05/2020). The simulations ended on day 105 (31/05/2020). We calibrated the model during the three phases (before, during, and after the intervention) and interpreted the results. Then, we fixed the parameters with minimal contribution to the model uncertainty to their nominal values and performed another set of simulations up to Dec 2020 (MC2: 15/02/2020–15/12/2020). The ambition was to investigate the existence of a significant climatic signal, diagnostically evaluate the transmission dynamics in a temperate climate during the spring and autumn waves, quantify the controlling factors and assess the variability in forecast uncertainty.

### 3.1. Environmental Clustering of COVID-19 Data

**Figure 3:**
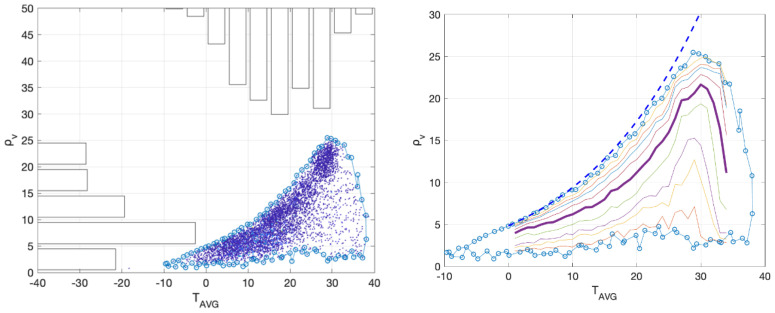
Temperature and humidity conditions for all reported COVID-19 human cases worldwide (01/01/2020–07/05/2020). In the same figure, univariate histograms of the scattered-data are plotted. The frequency of the joint distribution is given in Table 2 [left]. Percentiles (1, 5, 10, 25, 50, 75, 90, 95, 99) of humidity as a function of temperature for all reported COVID-19 human cases worldwide (01/01/2020–07/05/2020). The thickest line corresponds to the median. In the same figure, the maximum possible value of humidity at each temperature, estimated from the Clausius-Clapeyron equation, is plotted with the dotted line [right].

COVID-19 cases have been reported across a wide range of climatic conditions (Figure 3), ranging from −10 °C to 38 °C for temperature and from 0.8 g m^−3^ to 25 g m^−3^ for absolute humidity. For temperatures up to 25 °C, the absolute humidity reached its upper ceiling (saturation value) given by the Clausius-Clapeyron equation. In addition, half of the occurrence points were located close to the saturation curve for temperatures between 0 and 25 °C, suggesting that at this temperature range high relative humidity favors the persistence of SARS-CoV-19 and thus the emergence of COVID-19 cases. Therefore, there existed a tendency for more occurrences as the climate became cooler and more humid. Although temperature and humidity may influence virus inactivation in the environment, a causal mechanism has not been shown yet [58,59].

The most frequent humidity zone spanned the area 5–10 g m^−3^ (37.4%), while for temperature 71.3% of the reported cases were observed between 10 °C and 30 °C (Figure 3). With respect to their joint probability, the zone $\{5\leq \rho_{v}\left({g\;m}^{-3}\right)\leq 10,$ 10 ≤ *T*(°C) ≤ 20} had the highest frequency of COVID-19 cases which accounts for 22.8% of them. This classification had similarities with the findings of [43] who reported temperature in the range 5–11 °C and absolute humidity in the range $4–7\;{g\;m}^{-3}$ at cities with substantial transmission. This was not too different from our result, considering the uncertainty due to the different datasets (point measurements in our study versus gridded reanalysis). This result was also aligned with other studies, which indicated that a favorable area around 10 °C/5$\;{g\;m}^{-3}$ existed.

A series of measures was employed by different countries to reduce the contact rates including mobility restrictions, mask usage, school and business closures. The public health practices of governmental interventions such as quarantine and isolation of individuals and the timing of initiation of a measure during the course of the epidemic varied substantially across countries. These factors controlled largely the transmissibility of SARS-CoV-2. Moreover, there are additional factors which may affect virus transmissibility such as population density and weather conditions. The combined effect of all contributing factors in the probability of infection occurrence and the number of human cases, at the early stages of the epidemic, is presented in Table 2. The variability of the (median) number of COVID-19 cases per million did not follow the same pattern with the probability of infection. The precise quantification of the impact of each factor is beyond the scope of this article. Assuming that weather conditions had a stronger effect than the intervention measures given similar populations (density, age structure), the magnitude of the outbreak would be comparable in countries with similar weather conditions, population density and age structure (e.g., Greece, Spain). This was not observed. Furthermore, in our analysis we reinitialized the simulations over a monthly time scale where the climatic signal was considered constant, hence they were excluded. Based on this simplified concept, we did not apply any climatic dependence in the transmission rates in our subsequent analysis.

### 3.2. Model Calibration for Greece: March-April-May 2020

**Figure 4:**
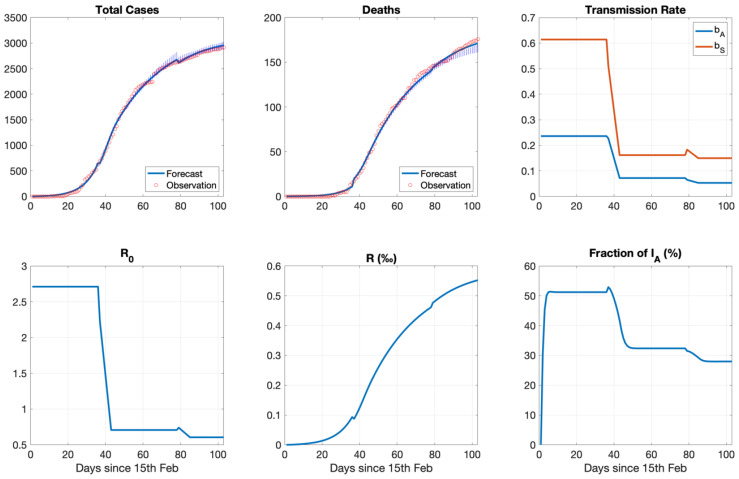
[Top row] Dynamic evolution of the ensemble of simulations (boxplot (light blue) with interquartile range (deep blue part), which set the uncertainty limits of the forecast, together with the optimal simulation (solid line) and the observations (red circles). Left panel shows the symptomatic infected persons (*I_S_*), middle panel the deaths, right panel the *R*_0_. [Bottom row] Optimum transmission rates identified from the MC experiments (left panel). Dynamic evolution of the corresponding fraction of the population who recovered (middle panel). Fraction of the asymptomatic infected persons (*I_A_*) among all infected cases (right panel). See text for explanation.

Model Validation: The best-fit analysis filtered out the 0.5% of the 20,000 MC simulations with the lower normalized mean square error (100 simulations). The single simulation with the lowest error was the optimum. When we superimposed the time series of cases with the best-fit analysis of the model for the infected persons with symptoms and the fatalities we observed an excellent result in terms of accuracy and phasing (Figure 4). In the same figure, the model uncertainty was also presented (box plot with IQR of the simulations falling below the 0.5th percentile with respect to the error). The best-fit analysis followed the cases as they occurred with slowly increasing uncertainty. Another model prediction that could be validated was the effective reproduction number. Initially, the basic reproduction number (*R*_0_) was around 2.7 and the subsequent effective reproduction number fell below the critical value of one after the intervention. The reproduction number remained below the critical value of one when the lockdown was lifted. Those numbers were in agreement with the reproduction number officially reported from the COVID-19 committee of Greece [60]. Overall, the experiment MC1 replicated the observed curves (total cases, deaths) with small uncertainty and also demonstrated good accuracy and phasing with the observed reproduction number curves.

Transmission: We assumed a difference in transmission rate between asymptomatic infected persons and persons with clinical symptoms [9]. The posterior distribution after the best-fit analysis for the transmission rate between an asymptomatic and a susceptible (*β_A_*) had an optimal value of 0.24/day. The corresponding value of the transmission rate between an infected symptomatic and a susceptible (*β_S_*) was 0.61/day and thus 2.5 times higher than that between asymptomatic infected individuals and susceptibles in the optimal case. After the intervention measures, the transmission rate of an infected symptomatic dropped to 0.16/day, i.e., close to the level of *β_A_* prior to the intervention. The transmission rate between asymptomatics and susceptibles dropped to 0.07/day. The relation *β_S_*/*β_A_* remained similar during the intervention. When the lockdown was lifted, *β_S_* and *β_A_* dropped further to 0.15/day and 0.05/day. See also Table 3.

Incubation period and asymptomatic infections: The incubation period has been estimated to have a mean value of 5.8 days (95% CI 5.0–6.7) [61]. Before the implementation of social distancing (lockdown), susceptible individuals became exposed and stayed in the latent phase of being infected and not infectious for 1.9 days (IQR 1.9–2.1). Asymptomatic infected individuals before they progressed to the symptomatic state remained asymptomatic for 3.6 days (IQR 3.6–4.1) and had an incubation period of 5.5 days (IQR 5.5–6.1). During the intervention asymptomatic individuals before progressing to the symptomatic state had an incubation period of 6 days (IQR 5.6–6.4) which dropped to 5.8 days (IQR 5.7–6.5) in the epoch after the lockdown. (Table 3).

Recovery: Asymptomatic infected persons were assumed to have a higher recovery rate than persons with clinical symptoms and thus a shorter recovery period. Asymptomatic infected individuals remained asymptomatic for the duration of their infectiousness and recovered after 4.4 days (IQR 3.8–4.5), 4.5 days (IQR 3.8–4.5), 3.8 days (IQR 3.7–4.2) before, during and after the intervention respectively. For asymptomatic infected individuals who remained asymptomatic (preclinical) before developing symptoms the corresponding values were 3.6 days (IQR 3.6–4.2), 4 days (IQR 3.7–4.3), 3.7 days (IQR 3.7–4.3). Symptomatic infected persons had an optimal recovery rate of 0.15/day which corresponds to a duration of recovery of 6.7 days (IQR 6.7–7.7) after onset of symptoms with very similar values during and after the intervention. The proportion of deaths was 4.1% (IQR 4.1–4.6) before the lockdown and 5.7% (5.5–5.8) during the lockdown with similar proportion after the lockdown 5.9% (IQR 5.6–6.0). See also Table 3.

**Figure 5:**
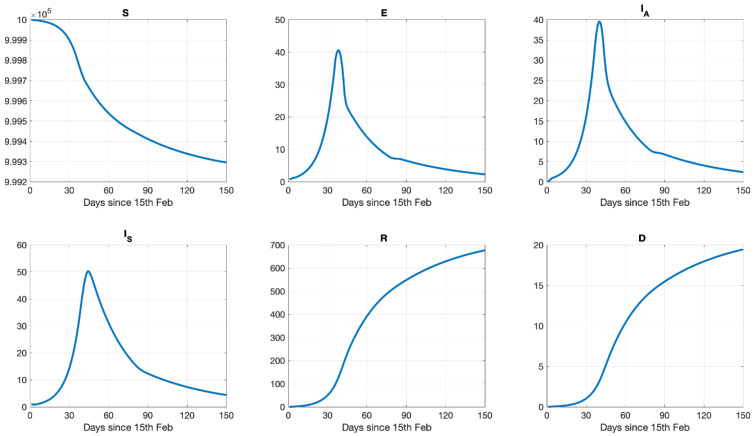
Model population dynamics (per million) with the optimal configuration for Susceptibles (*S*), Exposed individuals (*E*), Asymptomatic infected individuals (*I_A_*), Infected individuals with symptomatics (*I_S_*), Recovered (*R*), Dead (*D*).

Dynamics: Figure 5 shows the dynamics of the different populations, susceptible persons, asymptomatic infected persons, symptomatic infected persons, those who recovered, and the fatalities including the interventions, which were introduced on day 37 in our simulation of the COVID-19 epidemic in Greece and were gradually removed from day 79 onwards. Susceptibles declined gently after the lockdown. Exposed reached a maximal value at the day of the intervention (day 37) and then dramatically declined, reaching asymptotically lower values after 90 days. Asymptomatic infected (*I_A_*) and individuals with clinical symptoms (*I_S_*) reached a maximal value that was interrupted by the introduction of the interventions leading to a permanent decrease of their population with a short time delay between *I_A_* and *I_S_*. The intervention period changed the curvature of the recovered (*R*) and the fatalities (*D*) populations.

Asymptomatic fraction-Recovery: Besides the predictions that can be validated, there were at least two important model estimates, which, however, due to lack of appropriate data, could not be validated. The fraction of the infected asymptomatic individuals within the total population of infected individuals was estimated at 52% at the beginning of the epidemic and dropped to 33% one month after the intervention (lockdown). The first estimate for the asymptomatic fraction is in line with recent estimates about the attribution of at least 50% of COVID-19 infections to individuals that show no symptoms for a certain period but progress to onset of symptoms or they never show symptoms until they recover [62]. Whether the asymptomatic fraction declines during interventions would need observational confirmation though. The fraction of the population that had recovered at day 90 was estimated to be only 0.055% (Figure 4).

### 3.3. The Variation of Transmissibility: From March to December 2020

The results of the previous section suggest that we may fix all parameters except the transmission rate between a symptomatic and a susceptible individual (*β_S_*). Therefore, we kept all factors but *β_S_* fixed and performed another cycle of Monte Carlo simulations where we sought the intra-annual variability of the optimal *β_S_*. We split the period 15/02/2020–15/12/2020 into temporal windows of equal size and calibrated the model within each period following a slightly different approach than before for reasons explained hereafter. The COVID-19 tests performed weekly varied from 7000 in March-April to 65,000 in July–August to 130,000 in September–October 2020. After August, the total number of weekly tests included a portion of rapid tests having lower sensitivity and specificity than polymerase chain reaction (PCR) tests. Moreover, the sampling gradually included an increasing number of random tests. To get over with those uncertainties as we do not know the number of asymptomatic individuals detected daily nor the implemented sampling strategy, the calibration for the whole period was based on the number of deaths, which were not affected by the abovementioned uncertainties.

**Figure 6:**
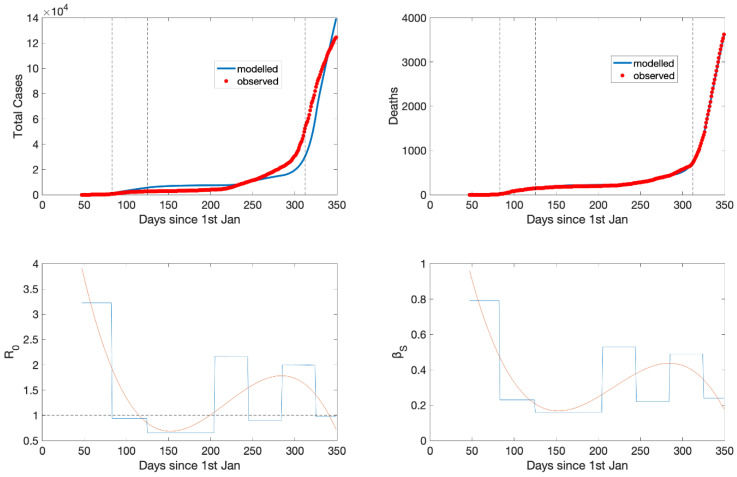
Dynamic evolution of the simulation with optimal transmission rate (*β_S_*) at each time block. [top row] modelled number of symptomatic infected persons (*I_S_*) (blue, left panel) and deaths (blue, right panel) together with the observed number of positive cases (red circles) and the observed number of deaths (red circles). [bottom row] Modelled *R*_0_ (blue, left panel) and *β_S_* (blue, right panel) together with the polynomial fit (right). See text for explanation.

The modelled and observed curves of the deaths almost coincided (Figure 6). For the infected cases, the agreement was also good considering that they were not used at all in the calibration process. Moreover, the underestimation in the most recent period may be attributed to the increased number of tests performed. In addition, the polynomially fitted reproduction number profile replicated exactly the observed reproduction number in Greece, which was below one between mid-April and last week of July and fell below one again in the second week of December. This is aligned with the variation of *β_S_* which exhibits three discrete peaks: two related with the timing of the spring and autumn epidemic waves and another one in between.

Τhe precise replication of the time evolution of deaths, which was not affected by the tests performed, and especially the replication of the observed reproduction number curve constitute a robust indicator for the model fit and the estimated *β_S_* which we analyzed next in predictive mode.

### 3.4. The Variation of Predictability: From March to December 2020

The results in Section 3.2 and Section 3.3 provided diagnostic estimates of the transmission rates, yielding constant values over a fixed time frame spanning few weeks. We investigated the daily variability of the transmission rates in predictive mode and their relation to the forecast horizon. We used moving windows of size 25 days (3.5 weeks) and we estimated the *β_S_* that optimized the error, as performed before. Then, using the optimal *β_S_*, we continued the simulation for 30 days beyond the last day of the window (*T_E_*) and we searched for the day after *T_E_* when the NRMSE exceeded 0.1 (i.e., the forecast error becomes 10%).

**Figure 7:**
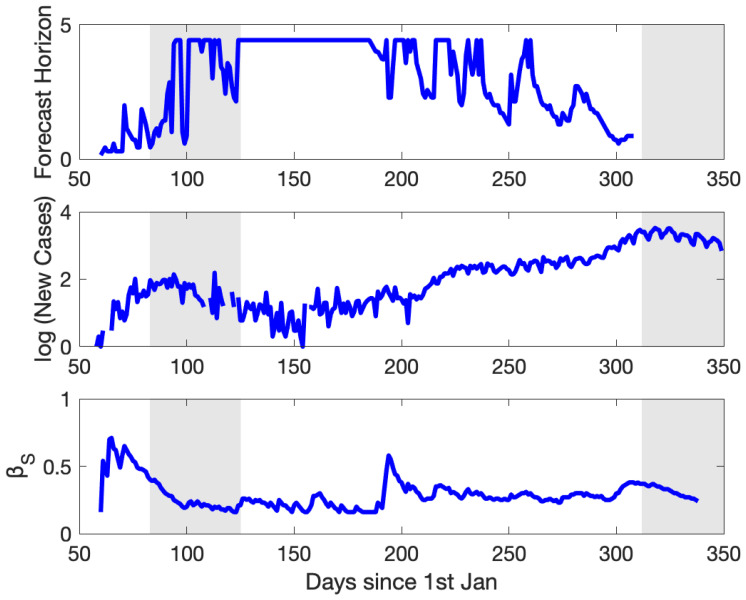
The forecast horizon and its variability (top panel), together with the evolution of the new cases (middle; from observations) and the *β_S_* (bottom panel). The lockdown periods are shaded. See text for explanation.

The diurnal variation of the *β_S_*, closely replicated the coarse pattern seen earlier using non-moving temporal windows (Figure 7), implying the internal dynamics were invariant under different model configurations. Three peaks were evident in *β_S_* occurring with decreasing magnitude; apart from the spring (strongest: 0.71/day) and autumn (weakest: 0.38/day) signals, there existed an intermediate peak at the beginning of July (0.58/day). The transmission rate initially decreased gradually following the intervention measures and reached a plateau. The abrupt increase in the *β_S_* at the beginning of July was probably related to the relaxation of social distancing measures such as the maximum number of participants in social events and the opening of the touristic season, which occurred few weeks earlier. The change was also evident at the curve of the new cases. The forecast horizon depended on the *β_S_* magnitude and its day-to-day variability. Large and variable *β_S_* resulted in smaller forecast horizons. The same held true for the upslope portion of the epidemic curve, where the fastest changes were linked to the least predictability. In the case of the Greek data, the implemented error tolerance resulted in a minimum forecast horizon of roughly one week, observed twice during the periods of the steepest change in the epidemic curve (March, November). At the opposite end, the forecast horizon exceeded four weeks during the period April–July when the variability was smoother. Therefore, the current epidemiological state is a significant indicator of the length of time into the future over which the forecasts are reliable [63].

The predictability horizon for models with sensitive dependence on initial conditions can be extended with ensemble methods, typically employed in weather prediction [64] and weather-dependent coupled-systems [65], to provide skillful forecasts in the region between divergent solutions and error saturation. The existence of accurate data is among the requirements of the framework.

## 4. Discussion

Aim of this study was the exploration of the transmission dynamics of SARS-CoV-2 in a temperate climate and its potential predictability. The role of asymptomatic infections and the potential effects of climatic factors in the epidemiology of COVID-19 were also investigated. We used an epidemiological model that explicitly included the dynamics of the asymptomatic infected population and allowed for a quantification of its relation to the population of infected person showing clinical symptoms among other aspects. Using a Monte Carlo uncertainty framework, we applied the model in the case of the COVID-19 epidemic in Greece including the imposed public health interventions of social distancing, diagnostically evaluated the model parameters and quantified the controlling factors.

Taking into account the range of climatic conditions in terms of temperature and humidity where COVID-19 cases occurred, we identified the temperature and humidity ranges in which COVID-19 cases may appear with increased probability and with increased number of cases. The inconsistency between the two maps indicate that climatic factors may not be of dominant importance for COVID-19 transmission.

The analysis of the modelling results at the intra-annual scale allowed for in depth investigation of several scenarios that included the transmission dynamics during the 15 Feb. and 15 Dec. 2020 and the importance of the model parameters. Moreover, the analysis at this scale permitted the exploration of the forecast horizon and its variability. Out of the seven model parameters, all but the transmission rates could be fixed throughout the investigated period. Three discrete peaks were found in the transmission rates. Two of them corresponded to the timing of the epidemic waves (spring and autumn respectively) while the third one occurred in mid-summer, implying that relaxation of social distancing and increased mobility may substantially influence the epidemic dynamics during summer and lead to a resurgence of infections offsetting positive effects from factors such as decreased household crowding indoors and environmental ultraviolet radiation. Notably, although transmission rates in autumn were estimated to be lower than in spring the epidemic dynamics were much stronger in autumn probably due to the advanced spread of the infection in the population.

The transmission rate from a symptomatic infected person to a susceptible compared to that of an asymptomatic infected person was substantially higher. Changes in the transmission rate followed the pattern of the introduction and relaxation of public health interventions. Magnitude and variability of the transmission rates determined the length of the forecast horizon. Moreover, the actual epidemiological activity was critical for the predictive reliability of the forecast horizon in time. In our case this period spanned from as low as few days (less than one week) to more than four weeks. Transmission strongly depends on viral load and viral shedding. In some studies of COVID-19 patients viral loads in the upper respiratory tract were found to be similar between symptomatic infected and asymptomatic infected persons indicating a higher than expected transmissibility for the asymptomatic population group. It seems, however, that clinical symptoms of infected persons such as increased viral shedding due to more frequent expiratory events like sneezing, coughing makes a difference in transmissibility. In general, the population group specific transmissibility has been an open question and has to be further explored in clinical and experimental studies.

The differences in recovery rates between asymptomatic infected and symptomatic infected persons have been observed and confirmed in our numerical results. Moreover, we provided optimal values for the incubation period and the residence time in the preclinical and clinical states.

The fraction of asymptomatic cases before and after the epidemic control interventions could be quantified and contributes to the understanding of the changes over time of this critical subpopulation. The attribution of a large part of infections to exposure to asymptomatic infected individuals, which have rather lower transmissibility compared to infected individuals with clinical symptoms and their contribution to the transmission dynamics has to be better assessed. SARS-CoV-2 transmission from subclinically infected persons is critical for public health decision making. There are some indications that pathogen transmission from this group of persons may be substantial implying that the incidence of asymptomatic compared with symptomatic SARS-CoV-2 infections needs to be determined. Serological studies, surveillance, and testing can help in the identification of the extent of the infections attributable to asymptomatic infected individuals.

The limitations of our modelling results related to the case study were predominantly the underlying assumptions we made and the accuracy of the official data to which we applied the approach. For instance, the fraction of fatalities we found was consistent with observations. However, as in every epidemic the estimation of the true case fatality risk during the outbreak is extremely difficult due to a substantial number of unreported cases among other reasons such as delays in diagnosis and reporting, and observations probably overestimate the true case fatality rate [17]. The observed patterns of climatic conditions in temperate regions for respiratory viruses’ activity such as influenza and SARS-CoV-2 have been under investigation and attribution to specific environmental factors has been very difficult. Well-designed observational and experimental studies are needed to identify causality and minimize confounding. Moreover, immunological aspects such as changes in population immunity and virus transmissibility during the course of an epidemic and between seasons have to be better understood [40]. Pathogen and host specific effects related to virus survival and immunity respectively were also not considered here.

## 5. Conclusions

The approach presented here may be a useful tool for the study of the potential role of specific epidemiological sub-populations on the transmission dynamics of respiratory infectious diseases such as COVID-19. The model is expandable and can provide insightful quantification of the corresponding effects pointing to the relative importance of other factors that may influence contact rates, virus inactivation and immunity and thus affect pathogen transmission. The model can be embedded in an ensemble framework to extend the predictability horizon, provided accurate and/or representative observations are available. The temporal lengthening of the early warning information should be of relevance for the design of targeted public health responses, especially in the low-predictability epochs before outbreaks.

## Figures and Tables

**Table 1:** Prior Distributions based on a uniform distribution U (min, max) during the screening phase and normal distribution N (*μ*, *σ*) during the implementation phase. Transmission rate (*β_S_*) values refer to those before, during and after the intervention (lockdown).

		Screening Phase		Implementation Phase	
Parameter	Description	Min	Max	*μ*	*σ*
*α*	Latency rate (day^−1^)	0.3	5	0.5	0.03
*β_S_*	Transmission rate between *I_s_* and *S* (day^−1^)	0.3	1.5	0.6	0.03
				0.2	0.03
				0.2	0.03
*μ* = *β_A_/β_S_*	Transmission rate ratio (day^−1^)	0.2	1	0.35	0.037
*γ_A_*	Recovery rate from subclinical infection (day^−1^)	0.07	0.5	0.25	0.03
*γ_S_*	Recovery rate from clinical symptoms (day^−1^)	0.05	0.2	0.15	0.02
*δ*	Transition rate at which *I_A_* becomes *I_S_* (day^−1^)	0.07	0.5	0.25	0.05
*f_S_*	Deaths (%)	0.01	0.09	0.05	0.005

**Table 2:** Probability of infection (estimated from event occurrence) and human cases per million (estimated from detected cases) with respect to temperature and absolute humidity, based on reported COVID-19 human cases worldwide (01/01/2020–07/05/2020). Shaded cells contain more than 5% of the data.

	Probability of Infection (%)					Cases per Million (Median)				
*ρ_v_*\T_AVG_	−10–0	0–10	10–20	20–30	30–40	−10–0	0–10	10–20	20–30	30–40
**0–5**	17	38	72	10	24	3.6	11.7	10.8	0.6	0.8
**5–10**		32	50	51	70		3.1	5.0	1.7	2.3
**10–15**			52	35	77			2.3	1.2	0.9
**15–20**			29	25	58				1.4	0.6
**20–25**				28	37				1.1	1.8

**Table 3:** Posterior distributions of the optimum model parameters (1/day). The numbers in parenthesis denote the interquartile range (IQR) of the 0.5th percentile. Values before, during and after the intervention (MC1: 15/02/2020–31/05/2020). The values in the last column are used in the MC2 runs (15/02/2020–15/12/2020).

Parameter	MC1			MC2
	Before Intervention	During Intervention	After Intervention	
*α*	0.52 (0.48 0.52)	0.50 (0.48 0.52)	0.47 (0.47 0.50)	0.52
*β_S_*	0.61 (0.59 0.62)	0.16 (0.16 0.19)	0.15 (0.14 0.20)	
*β_A_*	0.24 (0.22 0.24)	0.07 (0.07 0.08)	0.05 (0.05 0.07)	*β_S_*/3
*γ_A_*	0.23 (0.22 0.26)	0.22 (0.22 0.26)	0.26 (0.24 0.27)	0.24
*γ_S_*	0.15 (0.13 0.15)	0.15 (0.14 0.16)	0.15 (0.15 0.16)	0.15
*δ*	0.28 (0.24 0.28)	0.25 (0.23 0.27)	0.27 (0.23 0.27)	0.25
*f_S_* (%)	0.041 (0.041 0.046)	0.057 (0.055 0.058)	0.059 (0.056 0.060)	0.03

## Data Availability

Data will be made available from the authors upon request.
